# Food for the Sick, and How to Prepare It

**Published:** 1900-12

**Authors:** 


FOOD FOR THE SICK, AND HOW TO PREPARE IT.—With a chapter on food for the baby. By Edwin Charles French, M. D. 171 pages. Price, $1.00. Louisville: John P. Morton & Co. 1900.

This little book is reliable, and its contents should be familiar to all up-to-date physicians. It is a correct statement to make that the tendency of medicine at this time is more in the direction of the nutritional than the medicinal; therefore, a resume of scientific knowledge on the subject of food for the sick is eminently timely and important. T. J. B.

